# Post percutaneous coronary interventional adverse cardiovascular outcomes and bleeding events observed with prasugrel versus clopidogrel: direct comparison through a meta-analysis

**DOI:** 10.1186/s12872-018-0820-6

**Published:** 2018-05-02

**Authors:** Pravesh Kumar Bundhun, Feng Huang

**Affiliations:** 1 Institute of Cardiovascular Diseases, the First Affiliated Hospital of Guangxi Medical University, Nanning, Guangxi 530021 People’s Republic of China; 2 Institute of Cardiovascular Diseases and Guangxi Key Laboratory Base of Precision Medicine in Cardio-cerebrovascular Diseases Control and Prevention, the First Affiliated Hospital of Guangxi Medical University, Nanning, Guangxi 530021 People’s Republic of China

**Keywords:** Prasugrel, Clopidogrel, Percutaneous coronary intervention, Bleeding events, Major adverse cardiac events

## Abstract

**Background:**

Due to limitations associated with clopidogrel following percutaneous coronary intervention (PCI), other newer oral anti-platelet agents are being studied. We aimed to systematically carry out a direct comparison of outcomes observed with prasugrel versus clopidogrel following PCI.

**Methods:**

Common online searched databases (The Cochrane library, EMBASE, MEDLINE and Google scholar) were used to retrieve relevant publications. Primary endpoints were the adverse cardiovascular outcomes. Secondary outcomes were the bleeding events. This analysis was carried out by RevMan 5.3, whereby odds ratios (OR) and 95% confidence intervals (CI) were considered as the statistical parameters.

**Results:**

Eight studies with a total number of 18,122 participants were included in this direct analysis. Prasugrel was associated with significantly lower adverse cardiovascular outcomes in comparison to clopidogrel following PCI. All-cause mortality, myocardial infarction, stroke, stent thrombosis and major adverse cardiac events were all significantly lower with prasugrel (OR: 0.47, 95% CI: 0.35–0.63; *P* = 0.0001), (OR: 0.68, 95% CI: 0.57–0.80; *P* = 0.00001), (OR: 0.60, 95% CI: 0.38–0.96; *P* = 0.03), (OR: 0.46, 95% CI: 0.30–0.72; *P* = 0.0006) and (OR: 0.61, 95% CI: 0.53–0.70; *P* = 0.00001) respectively.

When the bleeding outcomes were analyzed, Thrombolysis in Myocardial Infarction (TIMI) defined major and minor bleeding were not significantly different (OR: 0.91, 95% CI: 0.66–1.27; *P* = 0.59) and (OR: 1.16, 95% CI: 0.85–1.59; *P* = 0.35) respectively. However, the combined ‘all bleeding events’ was significantly higher with prasugrel (OR: 1.32, 95% CI: 1.03–1.70; *P* = 0.03), but when patients with STEMI and those undergoing elective PCI were separately analyzed, no significant difference in overall bleeding was observed.

**Conclusion:**

Adverse cardiovascular outcomes were significantly lower with the use of prasugrel in comparison to clopidogrel following PCI. In addition, TIMI defined major and minor bleeding were not significantly different showing prasugrel to be well-tolerated following PCI especially in patients with acute coronary syndrome.

## Background

The new anti-platelet agent prasugrel has been approved for use after percutaneous coronary intervention (PCI). Ending of the year 2017 has witnessed many research articles still focusing on anti-platelet agents following PCI [1, 2]. Due to limitations associated with clopidogrel, other newer oral anti-platelet agents which could potentially replace clopidogrel are presently still being studied.

Several recently published meta-analyses comparing prasugrel with clopidogrel are available in MEDLINE and other electronic databases. However, when those research were deeply assessed, several limitations were observed. To be clear, almost all of the meta-analyses compared clopidogrel versus a combination of prasugrel and ticagrelor [3]. For example, in 2013, a meta-analysis compared newer potential anti-platelet agents (prasugrel and ticagrelor combined together) with clopidogrel following PCI [4]. Similarly in 2015, another meta-analysis involving only 4 randomized controlled trials again compared newer oral P2Y12 inhibitors (prasugrel and ticagrelor) with clopidogrel [5].

Because data on this aspect were limited, indirect comparisons were also made through network meta-analyses [6]. Fortunately, one meta-analysis at least compared clopidogrel with prasugrel (without the inclusion of other newer oral P2Y12 inhibitors) [7]. However, a major shortcoming was the fact that a detailed comparison of bleeding events and other adverse cardiovascular outcomes were not carried out.

Therefore, in this analysis, we aimed to systematically carry out a detailed direct comparison of outcomes observed with prasugrel versus clopidogrel following PCI.

## Methods

### Searched databases and searched strategies

Common online searched databases (The Cochrane library, EMBASE, MEDLINE and Google scholar) were used to retrieve relevant publications.

Official websites of several cardiology journals were also carefully checked for relevant publications.

The following searched terms were used to retrieve English publications:

Prasugrel and clopidogrel; Oral P2Y12 inhibitors and clopidogrel; Prasugrel and percutaneous coronary intervention; Prasugrel and coronary angioplasty; Clopidogrel, prasugrel and percutaneous coronary intervention; Clopidogrel, prasugrel and PCI; Clopidogrel, prasugrel and acute coronary syndrome/ACS.

The above-mentioned searched databases were filtered for relevant publications, and those which were found important were at first saved in a specific folder to be further reviewed based on the inclusion and exclusion criteria.

### Inclusion and exclusion criteria

Selection of relevant publications was based on the following inclusion criteria:

- Randomized controlled trials or observational studies; (b) Comparing prasugrel versus clopidogrel following PCI; (c) Reporting adverse cardiovascular and bleeding outcomes among their clinical endpoints.

Publications were reviewed and then rejected based on the following exclusion criteria:

- They were non-English publications (it should be noted that non-English publications were excluded since the authors would not be able to fully understand the language and important information would be ignored or missed and inappropriate data would be included); (b) Case-control studies, review articles, or letter to editors; (c) The studies involved patients who were not undergoing PCI; (d) They compared prasugrel with clopidogrel, however, PCI was not involved; (e) They did not report adverse cardiovascular and bleeding outcomes; (f) They were duplicated studies; (g) They involved switching from clopidogrel to prasugrel or vice-versa (crossing over).

### Types of participants, outcomes, definitions and follow-ups

Participants who underwent PCI were included in this analysis: patients with acute coronary syndrome (ACS) especially ST segment elevated myocardial infarction (STEMI) or patients who were candidates for elective PCI were included.

The primary endpoints were the adverse cardiovascular outcomes:

All-cause mortality; Cardiovascular death; Myocardial infarction (MI); Stroke; Stent thrombosis; Repeated revascularization; Major adverse cardiac events (including death, MI, revascularization/stroke).

The secondary endpoints were the bleeding outcomes:

‘All bleeding events’ was defined as any possible type of bleeding which was reported; Thrombolysis In Myocardial Infarction (TIMI) defined major bleeding [8]; TIMI defined minor bleeding [8].

The types of participants, the outcomes which were reported in each study as well as the follow-up periods were listed in Table 1.

**Table 1:** Reported outcomes, types of participants and follow ups

Trials	Outcomes	Follow-up periods	Types of participants
INFUSE AMI [11]	All-cause death, MACEs, definite and probable ST, HORIZONS major bleeding	30 days and 1 year	STEMI undergoing PCI
JUMBO TIMI 26 [12]	Non-CABG TIMI major and minor bleeding, minimal bleeding, MACEs, all-cause death, stroke, MI, recurrent ischemia, ST	30 days	Elective or urgent PCI
PRASFIT ACS [13]	MACEs, all-cause death, CV death, ST, revascularization, MI, stroke, TIMI defined minor and major bleeding	6 months, 1 year	ACS undergoing PCI
PRINCIPLE TIMI 44 [14]	TIMI major and minor bleeding, overall bleeding events, ST, MI, MACEs	1 month	Elective PCI
TAILOR [15]	MACEs, MI, stroke, ST, CV death	1 month, 1.5 years	Elective PCI
TRANSLATE ACS [16]	All-cause death, MI, stroke, revascularization, MACEs, BARC bleeding type 1–5, GUSTO moderate/severe bleeding	6 months	ACS undergoing PCI
TRIGGER PCI [17]	MACEs, CV death, MI, definite and probable ST, all-cause death, TIMI major bleeding	8 months	Elective PCI
TRITON TIMI 38 [18]	MACEs, CV death, all-cause death, MI, stroke, revascularization, ST, TIMI major or minor bleeding	15 months	STEMI undergoing PCI

*Abbreviations*: *MACEs* major adverse cardiac events, *HORIZONS* Harmonizing Outcomes with RevascularIZatiON and Stents in Acute Myocardial Infarction, *CABG* coronary artery bypass grafting, *TIMI* thrombolysis in myocardial infarction, *ST* stent thrombosis, *CV* cardiovascular, *MI* myocardial infarction, *GUSTO* Global Use of Strategies to Open Occluded Arteries, *BARC* bleeding defined according to the academic research consortium, *STEMI* ST elevated myocardial infarction, *PCI* percutaneous coronary intervention, *ACS* acute coronary syndrome, *CAD* coronary artery disease, *T2DM* type 2 diabetes mellitus

### Data extraction and quality assessment and statistical analysis

Two authors (PKB and FH) independently reviewed the searched databases and extracted data from relevant publications.

The following data were extracted from the studies:

Total number of participants who were treated by prasugrel and clopidogrel respectively; The baseline features and the types of participants; The loading and maintenance dosages of prasugrel and clopidogrel; The methodological qualities of the trials; The adverse cardiovascular and bleeding outcomes as well as the corresponding follow-up periods which were reported.

After data extraction, data were further cross-checked. Any disagreement was resolved by consensus.

The methodological quality of the trials were also assessed based on the criteria set by the Cochrane collaboration [9]. A grade ranging from A (lowest risk of bias) to E (highest risk of bias) was allotted to each trial based on the assessment.

This analysis was carried out by RevMan 5.3 (latest version), whereby odds ratios (OR) and 95% confidence intervals (CI) were considered as the statistical parameters.

Heterogeneity which is a common feature in meta-analyses, was assessed by:

(a) The Q statistic test whereby a *P* value less or equal to 0.05 was considered as statistically significant; (b) The I^2^ statistic test whereby heterogeneity increased with an increasing I^2^ value.

A fixed (I^2^ < 50%) or a random (I^2^ > 50%) effects model was used based on the I^2^ value which signified heterogeneity.

In addition, sensitivity analysis was carried out by a method of exclusion.

Publication bias was also assessed through funnel plots.

## Results

### Searched outcomes

The PRISMA guideline was followed [10]. This electronic search resulted in a total number of 2949 publications. After a careful assessment of the titles and the abstracts, 2794 irrelevant publications were eliminated.

Among the remaining 155 articles, further elimination was carried out (following a second review) based on the following criteria:

They were meta-analyses (7); They were review articles (4); They were case-control studies (3); They were letter to editors (3); They were not associated with PCI (2); They involved crossing over of drugs (switching from clopidogrel to prasugrel or vice versa) [8]; They reported platelet reactivity as outcomes (11); They were duplicated studies (109).

**Fig. 1:**
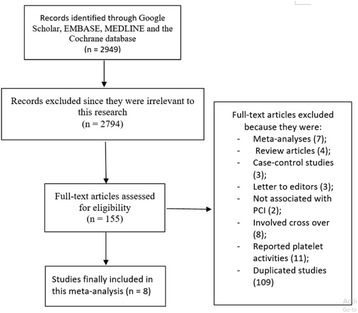
Flow diagram showing the study selection

Finally, 8 trials [11–18] were included in this analysis as shown in Fig. 1.

### General features of the studies

A total number of 18,122 participants were included in this direct analysis whereby 7051 participants were treated by prasugrel and 11,071 participants were treated by clopidogrel. The patients’ enrollment period varied from years 2003 and 2012. The general features of the studies were listed in Table 2.

**Table 2:** General features of the studies

Trials	Type of study	Time period of patients’ enrollment (years)	No of patients treated by prasugrel (n)	No of patients treated by clopidogrel (n)	Total no of patients (n)	Bias risk grade
INFUSE AMI	OS	–	155	297	452	B
JUMBO TIMI 26	RCT	2003	650	254	904	A
PRASFIT ACS	RCT	2010–2012	685	678	1363	B
PRINCIPLE TIMI 44	RCT	–	102	99	201	A
TAILOR	RCT	2010–2012	54	52	106	B
TRANSLATE ACS	OS	2010–2012	3424	7715	11,139	A
TRIGGER PCI	RCT	2009–2011	212	211	423	A
TRITON TIMI 38	RCT	2004–2007	1769	1765	3534	A
Total no of patients (n)			7051	11,071	18,122	

*Abbreviations RCT* randomized controlled trials, *OS* observational study

Based on the assessment of the methodological quality of the trials, a grade was allotted to each of the trials as shown in Table 2.

All the patients received aspirin along with a loading dose of either prasugrel (60 mg in most of the cases) or clopidogrel (300 or 600 mg) prior to PCI. Following PCI, aspirin and either prasugrel (10 mg) or clopidogrel (75 mg or 150 mg) were continually used during the follow-up periods as shown in Table 3.

**Table 3:** Loading and maintenance dosage of anti-platelet medications which were used by the participants

Trials	ASA administration throughout the study	GP IIb/IIIa	LD of prasugrel	LD of clopidogrel	MD of prasugrel	MD of clopidogrel
	P/C	P/C				
INFUSE AMI	100/100	+/−	–	–	10 mg	75 mg
JUMBO TIMI 26	100/100	+/−	60 mg	300 mg	10 mg	75 mg
PRASFIT ACS	100/100		20 mg	300 mg	3.75 mg	75 mg
PRINCIPLE TIMI 44	100/100	–	60 mg	600 mg	10 mg	150 mg
TAILOR	100/100	+/−	–	600 mg	10 mg	150 mg
TRANSLATE ACS	100/100	–	–	–	10 mg	75 mg
TRIGGER PCI	100/100	–	60 mg	600 mg	10 mg	75 mg
TRITON TIMI 38	100/100	+/−	60 mg	300 mg	10 mg	75 mg

*Abbreviations*: *P* prasugrel, *C* clopidogrel, *ASA* aspirin, *GP* glycoprotein, *LD* loading dose, *MD* maintenance dose

### Baseline features of the studies

The baseline features of the participants have been listed in Table 4. The average age varied from 56 years to 65.8 years. Male participants were predominant in both groups. The percentage of participants with risk factors such as hypertension, diabetes mellitus, dyslipidemia, smoking history have been listed in Table 4. According to the baseline features, there was no significant difference between participants who were treated by prasugrel or clopidogrel following PCI.

**Table 4:** Baseline features of the studies

Trials	Age (yrs)	Males (%)	HTN (%)	DSL (%)	DM (%)	CS (%)
	P/C	P/C	P/C	P/C	P/C	
INFUSE AMI	57.6/62.7	79.4/71.0	24.5/35.0	16.8/15.2	9.70/12.1	68.8/56.3
JUMBO TIMI 26	59.0/58.0	76.3/77.0	–	–	27.5/25.0	23.3/31.0
PRASFIT ACS	65.4/65.1	78.2/79.4	72.3/72.4	75.3/73.7	36.5/35.0	39.9/41.2
PRINCIPLE TIMI 44	64.0/63.8	71.6/77.8	85.3/77.8	90.2/86.9	32.4/29.3	17.6/16.2
TAILOR	63.0/63.0	74.1/82.7	74.1/82.7	83.3/88.5	29.6/34.6	77.8/67.3
TRANSLATE ACS	56.0/61.0	78.1/69.4	61.7/69.1	–	24.5/27.0	40.4/38.6
TRIGGER PCI	65.8/66.3	72.2/73.0	88.7/89.1	–	41.0/42.7	16.2/14.0
TRITON TIMI 38	58.0/59.0	79.0/76.0	49.0/50.0	41.0/41.0	19.0/19.0	47.0/44.0

*Abbreviations*: *yrs.* years, *P* prasugrel, *C* clopidogrel, *HTN* hypertension, *DSL* dyslipidemia, *DM* diabetes mellitus, *CS* current smoker

### Adverse cardiovascular outcomes observed with prasugrel versus clopidogrel following PCI

**Fig. 2:**
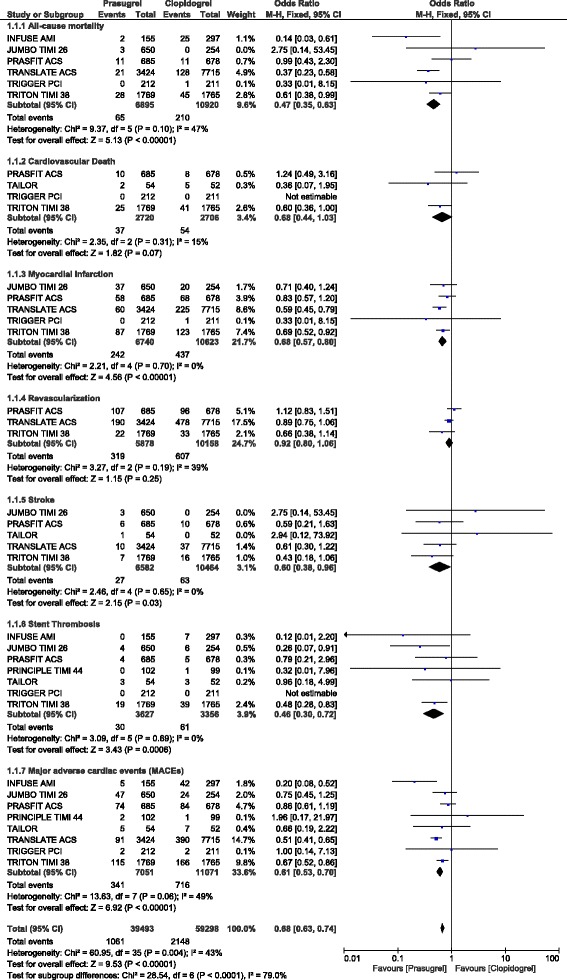
Adverse cardiovascular outcomes observed with prasugrel versus clopidogrel following PCI

Results of this analysis showed prasugrel to be associated with significantly lower adverse cardiovascular outcomes in comparison to clopidogrel following PCI. All-cause mortality, MI, stroke, stent thrombosis and MACEs were significantly lower with prasugrel (OR: 0.47, 95% CI: 0.35–0.63; *P* = 0.0001), (OR: 0.68, 95% CI: 0.57–0.80; *P* = 0.00001), (OR: 0.60, 95% CI: 0.38–0.96; *P* = 0.03, OR: 0.46, 95% CI: 0.30–0.72; *P* = 0.0006) and (OR: 0.61, 95% CI: 0.53–0.70; *P* = 0.00001) respectively as shown in Fig. 2. Repeated revascularization was not significantly different (OR: 0.92, 95% CI: 0.80–1.06; *P* = 0.25) (Fig. 2).

When participants which were extracted only from randomized controlled trials were assessed, all-cause mortality favored prasugrel (OR: 0.70, 95% CI: 0.47–1.05; *P* = 0.09), with a *P* value approaching statistical significance. Prasugrel was still associated with lower MI (OR: 0.73, 95% CI: 0.59–0.90; *P* = 0.003), stent thrombosis (OR: 0.49, 95% CI: 0.32–0.77; *P* = 0.002) and MACEs (OR: 0.74, 95% CI: 0.61–0.88; *P* = 0.0007).

The analysis which has been carried out above, included patients with STEMI undergoing PCI and other patients undergoing elective PCI.

**Fig. 3:**
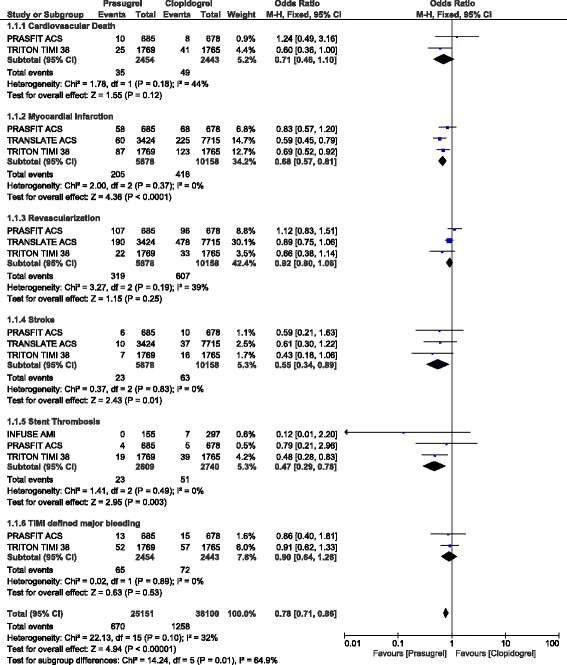
Adverse cardiovascular outcomes observed with prasugrel versus clopidogrel following PCI in patients with ACS

**Fig. 4:**
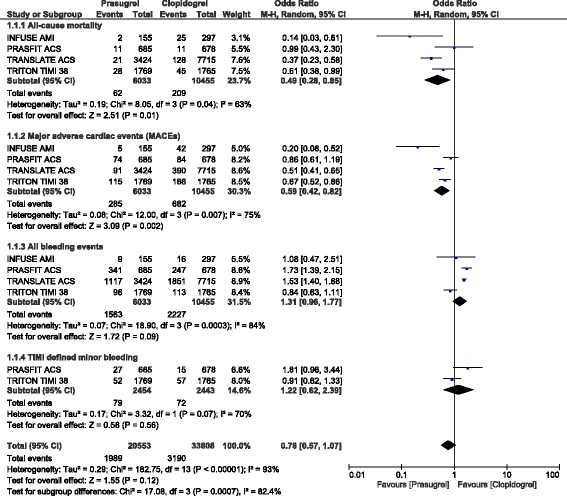
Adverse cardiovascular outcomes and bleeding events observed with prasugrel versus clopidogrel following PCI in patients with ACS

When patients with ACS (mostly STEMI) were separately analyzed, all-cause mortality, MI, stroke, stent thrombosis and MACEs were still significantly lower with prasugrel as compared to clopidogrel (OR: 0.49, 95% CI: 0.28–0.85; *P* = 0.01), (OR: 0.68, 95% CI: 0.57–0.81; *P* = 0.0001), (OR: 0.55, 95% CI: 0.34–0.89; *P* = 0.01), (OR: 0.47, 95% CI: 0.29–0.78; *P* = 0.003) and (OR: 0.59, 95% CI: 0.42–0.82; *P* = 0.002) respectively as shown in Figs. 3 and 4.

### Bleeding outcomes observed between prasugrel versus clopidogrel following PCI

**Fig. 5:**
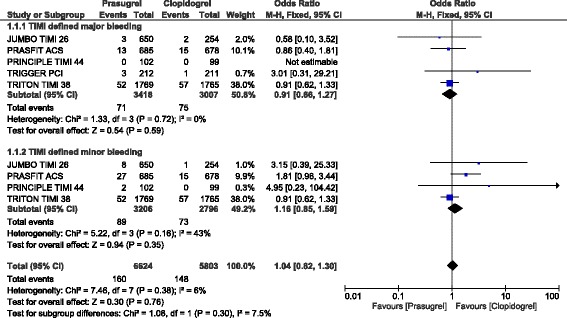
Bleeding events observed with prasugrel versus clopidogrel following PCI (part 1)

When the bleeding outcomes were analyzed, TIMI defined major and minor bleeding were not significantly different (OR: 0.91, 95% CI: 0.66–1.27; *P* = 0.59) and (OR: 1.16, 95% CI: 0.85–1.59; *P* = 0.35) respectively as shown in Fig. 5.

**Fig. 6:**
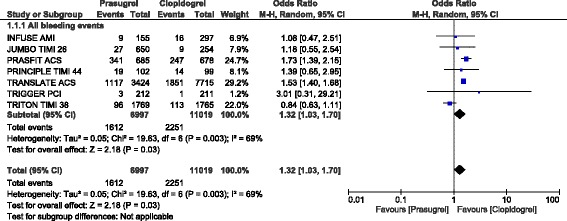
Bleeding events observed with prasugrel versus clopidogrel following PCI (part 2)

However, ‘all bleeding events’ was significantly higher with prasugrel (OR: 1.32, 95% CI: 1.03–1.70; *P* = 0.03) as shown in Fig. 6.

When we included only participants which were obtained from randomized controlled trials, TIMI defined major (OR: 0.91, 95% CI: 0.66–1.27; *P* = 0.59) and minor (OR: 1.16, 95% CI: 0.85–1.59; *P* = 0.35) bleedings were still similarly manifested with prasugrel and clopidogrel.

In patients with STEMI, TIMI defined major bleeding, and TIMI defined minor bleeding were also not significantly different (OR: 0.90, 95% CI: 0.64–1.26; *P* = 0.53) and (OR: 1.22, 95% CI: 0.62–2.39; *P* = 0.56) respectively as shown in Figs. 3 and 4. Even if ‘all bleeding events’ was significantly higher in these patients with STEMI who underwent PCI, (OR: 1.31, 95% CI: 0.96–1.77; *P* = 0.09), the result was not statistically significant as shown in Fig. 4.

**Fig. 7:**
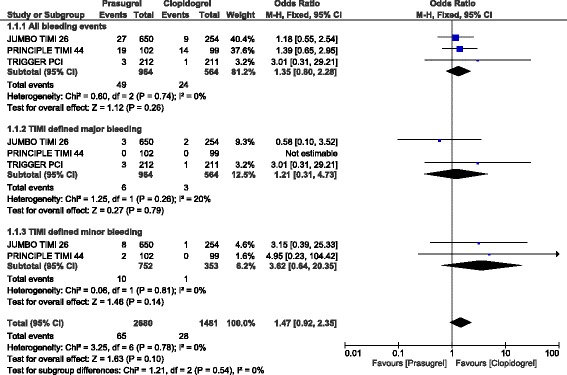
Bleeding events observed with prasugrel versus clopidogrel following elective PCI

In those patients without STEMI who underwent elective PCI, TIMI defined major, minor and ‘all bleeding events’ were not significantly different (OR: 1.21, 95% CI: 0.31–4.73; *P* = 0.79), (OR: 3.62, 95% CI: 0.64–20.35; *P* = 0.14) and (OR: 1.35, 95% CI: 0.80–2.28; *P* = 0.26) respectively as shown in Fig. 7.

Sensitivity analysis was also carried out without any significantly different result compared to the main analysis. Consistent results were obtained throughout all the subgroups.

### Publication bias

**Fig. 8:**
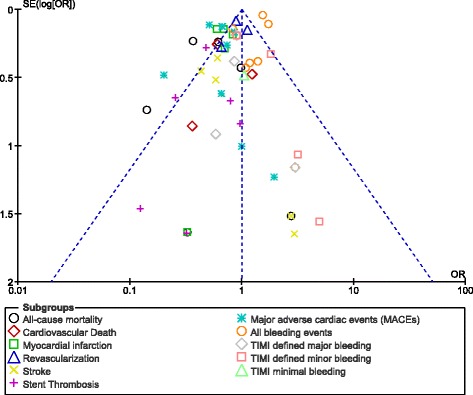
Funnel plot representing publication bias

This research analysis consisted of only eight studies. Funnel plots were most appropriate to represent publication bias for similar meta-analyses with a small number of studies. The funnel plot which was generated from Revman software showed low evidence of publication bias as shown in Fig. 8.

## Discussion

Prasugrel is a new anti-platelet agent which might potentially replace clopidogrel following PCI [19, 20]. In this analysis, a direct detailed systematical comparison was carried out between prasugrel and clopidogrel following coronary angioplasty.

The current results showed prasugrel to be significantly better than clopidogrel in terms of efficacy whereby mortality, MACEs, stroke and stent thrombosis were significantly reduced following PCI. TIMI defined major and minor bleeding were similarly manifested with prasugrel and clopidogrel. However, the combination of other types of bleeding which was defined as ‘all bleeding events’ was significantly higher with prasugrel, but when STEMI patients and those patients who underwent elective PCI were separately analyzed, no significant bleeding event was reported between prasugrel and clopidogrel.

This analysis showed robust results in comparison to another recently published meta-analysis [7]. Even though the current results were supported by the previous analysis (by Chen et al.), the latter involved several possible limitations. First of all, even though their analysis was based on patients post PCI, trials which did not involve patients who were revascularized by PCI were also included, contributing to its high total number of patients [21, 22]. In addition, trials which involved switching from clopidogrel to prasugrel or vice versa were also included [22]. Also, detailed outcomes were not assessed and the results which were reported showed an extremely high level of heterogeneity (up to 93%) in several subgroups [7]. The main strength of this current analysis was that it successfully resolved those flaws and limitations to provide a new analysis with robust results, with low heterogeneity in several of the subgroups, and reported a detailed analysis of the cardiovascular and bleeding outcomes.

Another analysis which was published on this aspect, involved a combination of prasugrel and ticagrelor which we think would be unfair to represent a result specifically based on prasugrel since it was associated with a combination of two anti-platelet agents [5].

In a Veterans Affairs population, prasugrel was proven to be significantly better compared to clopidogrel post PCI in terms of adverse cardiovascular events supporting the results of this analysis. Bleeding events were also not significantly higher [23].

In addition, a TRial to assess Improvement in Therapeutic Outcomes by optimizing platelet inhibitioN with prasugrel (TRITON)–Thrombolysis in Myocardial Infarction (TIMI) 38 Sub-study also showed results which supported this current analysis whereby prasugrel reduced ischemic events in comparison to clopidogrel [24]. Other studies have shown prasugrel to be even better than doubling the dosage of clopidogrel following stenting [25].

Furthermore, a retrospective study which compared prasugrel with clopidogrel in patients with ACS following coronary stenting also supported the current results showing significantly lower MI 1 year post PCI. The rate of minimal hemorrhage was higher with prasugrel but however, mortality was similar with both anti-platelet agents [26].

Another retrospective study showed prasugrel and clopidogrel to be similar in terms of adverse outcomes and bleeding events and the authors concluded that prasugrel was equally effective and safe [27]. This result was different from ours in terms of efficacy. But, the retrospective study was based on one single center whereas our current analysis involving mainly randomized patients from several larger trials, is believed to represent a better result.

However, the management of ACS does not only depend on anti-platelet agents. We should also emphasize on recent guidelines about how to manage patients with STEMI and NSTEMI. In this current analysis, more than 90% of the patients had STEMI whereas the remaining were NSTEMI patients. The 2017 ESC Guidelines for the management of acute myocardial infarction in patients presenting with ST segment elevation myocardial infarction gives a detailed explanation and treatment strategies of patients with STEMI [28] whereas the 2012 ACCF/AHA focused on the update incorporated into the ACCF/AHA 2007 guidelines for the management of patients with unstable angina and non-STEMI [29].

It should also be noted that diabetes mellitus and the associated medications might further influence the results of this analysis. In this analysis, each study included an average of 9.70 to 42.7% of patients with type 2 diabetes mellitus. However, a separate analysis of prasugrel versus clopidogrel in patients with diabetes mellitus was not possible because the trials did not report clinical outcomes and events separately for this particular subgroup of patients.

At last, it should be noted that incretin, a newer hypoglycemic drug, might also affect the prognosis of patients with diabetes mellitus and coronary artery disease. The effects of incretin treatment on cardiovascular outcomes in diabetic patients with STEMI [30] and NSTEMI [31] have previously been studied. However, in this current analysis, we were unable to assess the percentage of STEMI and NSTEMI patients on incretin treatment.

### Limitations

Even if the total number of patients were sufficient to reach a fair conclusion, several different subgroups reported less patients because the adverse cardiovascular outcomes and bleeding events were not reported in all of the trials; Different trials had different follow-up periods (1 month, 1 year to 5 years), and the follow-up periods were not taken into consideration; In addition, studies with planned PCI and ACS were mixed and analyzed at first; The other anti-platelet agents such as glycoprotein IIb/IIIa and double dose clopidogrel (150 mg instead of 75 mg), as well as the loading dose of prasugrel and clopidogrel which were different in certain studies might have influenced the results which were obtained; Moreover, publication bias was represented by funnel plots which were generated through the RevMan software. Any other additional statistical test was not carried out because the volume of studies was not sufficient.

## Conclusions

Adverse cardiovascular outcomes were significantly lower with the use of prasugrel in comparison to clopidogrel following PCI. In addition, TIMI defined major and minor bleeding were not significantly different showing prasugrel to be well-tolerated following PCI especially in patients with ACS. Future studies should be based on patients with diabetes mellitus.

## Abbreviations

ACS: Acute coronary syndrome
MACEs: Major adverse cardiac events
PCI: Percutaneous coronary intervention
TIMI: Thrombolysis in myocardial infarction
